# Antiparasitic Activity of Chalepensin and Graveoline Isolated from *Ruta chalepensis* L.: In Vitro Evaluation Against *Strongyloides venezuelensis*

**DOI:** 10.3390/pathogens14050419

**Published:** 2025-04-25

**Authors:** Nancy E. Rodríguez-Garza, Miguel Marín, Javier Sánchez-Montejo, Joel H. Elizondo-Luévano, Aldo F. Bazaldúa-Rodríguez, Ramiro Quintanilla-Licea, César I. Romo-Sáenz, Rafael Peláez, Antonio Muro, Julio López-Abán

**Affiliations:** 1 Grupo de Enfermedades Infecciosas y Tropicales (e-INTRO), Instituto de Investigación Biomédica de Salamanca, Centro de Investigación de Enfermedades Tropicales de la Universidad de Salamanca (IBSAL-CIETUS), Facultad de Farmacia, Universidad de Salamanca, 37007 Salamanca, Spain; 2 Departamento de Microbiología e Inmunología, Facultad de Ciencias Biológicas, Universidad Autónoma de Nuevo León, San Nicolás de los Garza 66455, Nuevo León, Mexico; 3 Departamento de Química, Facultad de Ciencias Biológicas, Universidad Autónoma de Nuevo León, San Nicolás de los Garza 66455, Nuevo León, Mexico; 4 Facultad de Medicina y Ciencias Biomédicas, Universidad Autónoma de Chihuahua, Chihuahua 31109, Chihuahua, Mexico; 5 Laboratorio de Química Orgánica y Farmacéutica, Departamento de Ciencias Farmacéuticas, Facultad de Farmacia, Universidad de Salamanca, 37007 Salamanca, Spain

**Keywords:** antiparasitic activity, chalepensin, graveoline, *Ruta chalepensis*, *Strongyloides venezuelensis*

## Abstract

Parasitic diseases constitute a significant challenge to global public health, with *Strongyloides stercoralis* ranking among the most prevalent and clinically significant parasites. The limitations of current nematocidal therapies highlight an urgent need for novel treatment strategies. In this study, the nematocidal activity of chalepensin and graveoline, two compounds isolated from *Ruta chalepensis*, was evaluated against larval and adult stages of *Strongyloides venezuelensis* (model for *S. stercoralis*). The in vitro efficacy of these compounds was assessed on third-stage infective larvae (L3) and adult parthenogenetic females at various time points, while cytotoxicity was determined using Vero cells to calculate selectivity indices (SI). Both compounds showed good antiparasitic activity, but chalepensin exhibited superior nematocidal activity compared to graveoline, with an LC_50_ of 3.9 µg/mL and an SI of 990 for L3, and an LC_50_ of 16.8 µg/mL and an SI of 200 for adult females at 72 h. Morphological analysis via scanning electron microscopy in adult females revealed that graveoline induced mostly cuticle detachment, while chalepensin caused protuberances across the parasite body. These findings suggest that both compounds possess promising antiparasitic potential, with chalepensin emerging as a particularly potent candidate for further exploration.

## 1. Introduction

Parasitic diseases pose a major global public health concern, particularly in tropical and subtropical regions where poor sanitary conditions facilitate their transmission [1]. Helminthic infections affect nearly a quarter of the world’s population, contributing significantly to disease burden and disability [2]. Among these infections, strongyloidiasis, caused by *Strongyloides stercoralis*, is one of the most neglected and challenging helminthiases to control and eradicate [3]. It is estimated to affect over 600 million people worldwide [4]. The infection begins when filariform larvae present in contaminated soil penetrate the host’s skin, subsequently migrating through the bloodstream to the lungs. From there, they ascend the respiratory tract, are swallowed, and reach the small intestine. There, they mature into adult females capable of reproducing via parthenogenesis [5]. Unlike most helminths, *S. stercoralis* has a unique autoinfective cycle that allows the parasite to persist within a host for decades, leading to chronic infections and posing significant therapeutic challenges [6]. Up to 70% of patients with chronic *S. stercoralis* infection may remain asymptomatic, with elevated blood eosinophil counts often serving as the only clinical indicator of strongyloidiasis [7]. In symptomatic cases, patients typically present with mild gastrointestinal and respiratory complaints, along with a characteristic skin rash known as *larva currens* [8]. Although chronic infections can be asymptomatic or exhibit mild symptoms, they carry the risk of progressing to hyperinfection syndrome, a severe, life-threatening disease with a reported fatality rate approaching 95% [9].

The World Health Organization (WHO) recommends ivermectin as the first-line treatment for strongyloidiasis due to its superior efficacy. In contrast, albendazole, which has comparatively lower efficacy, is considered a second-line therapeutic option [10]. However, high-quality evidence for the treatment of hyperinfection syndrome remains limited, and there have been reports of severe toxicities associated with high-dose ivermectin use [11]. For decades, ivermectin has been widely used in mass drug administration programs aimed at eliminating onchocerciasis and lymphatic filariasis. This extensive use has raised concerns about the potential emergence of drug resistance, an issue well-documented in veterinary medicine but not yet observed in human infections [10]. Given this potential threat, developing new therapeutic alternatives is needed.

The need for new antiparasitic therapeutic strategies has sparked growing interest in the use of natural products with antiparasitic activity. Several studies have demonstrated that plants exhibit promising nematocidal effects, opening new possibilities in the fight against helminths [12,13]. In a previous study, our research group evaluated the nematocidal activity of 12 ethnomedicinal Mexican plants against *S. venezuelensis* third-stage infective larvae (L3), with *Ruta chalepensis* standing out due to its potent activity [14]. *R. chalepensis* is a widely used plant in traditional medicine that has been studied for its attributed biological properties, including antimicrobial, antifungal, and antiparasitic activities [15]. The antiparasitic activity of this plant is reported against various protozoa, such as *Entamoeba histolytica* [16] and *Plasmodium berghei* [17], as well as helminths, including *Meloidogyne incognita*, *M. javanica* [18], *Teladorsagia circumcincta*, *Trichostrongylus* spp. [19], *Haemonchus contortus* [20] and *Echinococcus granulosus* [21].

Building on our previous findings, this study evaluated the in vitro nematocidal activity of two major compounds from *R. chalepensis*, graveoline and chalepensin, against *Strongyloides venezuelensis* (a widely used model for *S. stercoralis*) targeting both infective L3 and adult worms.

## 2. Materials and Methods

### 2.1. Plant Material and Isolation of Compounds

**Figure 1:**
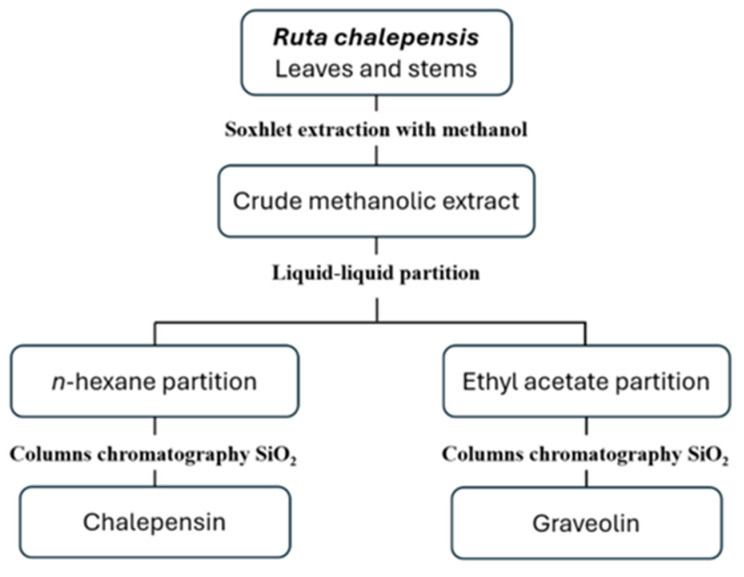
General diagram of isolation of chalepensin and graveoline from *Ruta chalepensis* leaves and stems.

Our working group previously published the isolation and identification of the compounds chalepensin and graveoline obtained from the leaves and stems of the plant *Ruta chalepensis* L. (voucher FCB-UANL 30654) [22,23,24]. Chalepensin was obtained from the *n*-hexane partition of *R. chalepensis* through column chromatography using silica gel 60 G (Merck, Darmstadt, Germany), while graveoline was isolated from the ethyl acetate partition, also by columns chromatography with silica gel 60 G (Figure 1). The compounds were identified through spectroscopy and spectrometry using a Bruker Spectrometer (Model Advance DPX400, 9.4 Teslas; Bruker Corporation, Billerica, MA, USA) and subsequently compared with existing literature data. The spectroscopic analysis data for the compounds can be found in the Supplementary Materials (Supplementary Materials_Spectroscopic Data).

### 2.2. Ethical Statement

All animal procedures adhered to the ethical guidelines outlined in Spanish regulations (RD 53/2013) and the European Union Directive (2010/63/CE) regarding the care and use of laboratory animals. The experiments were conducted at the accredited Animal Experimentation Facilities of the University of Salamanca (Registration No.: PAE/SA/001). The study protocols received approval from the Ethics Committee of USAL (CBE 335 CEI 1080). Efforts were consistently made to minimize animal suffering. The animals were housed in standard polycarbonate cages under controlled conditions, including a 12 h light–dark cycle, temperatures maintained between 23 °C and 25 °C, and unrestricted access to food and water.

### 2.3. Nematocidal Activity of Compounds Against Strongyloides venezuelensis

The *S. venezuelensis* strain, provided by the Department of Parasitology at the Federal University of Minas Gerais (Brazil), was maintained through successive passages in 4-week-old male Wistar rats (150–200 g) to ensure the completion of its life cycle. The rats were subcutaneously inoculated with 12,000 infective L3 suspended in 500 μL of phosphate-buffered saline (PBS). Fecal samples from rats were collected between days 5 and 18 post-infection and cultured in 250 mL polyethylene containers with vermiculite and distilled water. The cultures were kept in a humid environment at 28 °C for 4 to 7 days (SANYO Electric Co., Ltd., Gunma, Japan). L3 were subsequently isolated using the Baermann method, washed three times with distilled water, and their viability was confirmed under a light microscope before starting the experiments [14].

We placed between 100 and 150 L3 into each well of flat-bottomed 96-well microplates (Corning Incorporated) with 100 µL of distilled water. The larvae were incubated at 28 °C for 30 min to allow them to adapt. Subsequently, they were exposed to the test compounds at concentrations ranging from 1 to 50 µM. As controls, 10 µM ivermectin (Sigma-Aldrich, St. Louis, MO, USA) served as the positive control, while distilled water, with or without 1% DMSO, was used as the negative control. The larvae were incubated at 28 °C for 72 h under humid conditions (SANYO Electric Co. Ltd.), and mortality was assessed after 24, 48, and 72 h. Mortality was defined as the absence of movement, which was verified under a light microscope at 40× magnification for at least 1 min, using visible light to stimulate movement [14].

The parasitic females of *S. venezuelensis* live in the small intestine mucosa of an infected host and produce eggs by parthenogenesis [25]. To obtain the adult females, seven days after infection, the rats were euthanized by cervical dislocation after unconsciousness induced by carbon dioxide, and their small intestines were excised, opened longitudinally, finely chopped, and placed into a sedimentation cup lined with gauze in PBS. The samples were incubated at 37 °C for 2 h. Females adults were recovered from the sediment, washed twice with 0.85% saline solution, and then subjected to two additional washes in Roswell Park Memorial Institute 1640 medium (RPMI-1640) (Gibco, Whaltman, MA, USA) supplemented with 10% heat-inactivated fetal bovine serum (FBS), 100 µg/mL streptomycin, and 100 U/mL penicillin (Gibco, Whaltman, MA, USA) [26].

Subsequently, 50 to 70 parthenogenetic females were placed in 96-well plates containing RPMI-1640 medium and incubated at 37 °C for 1 h to allow for adaptation. Following this period, the test compounds were applied at concentrations from 1 to 60 µM. The anti-parasitic activity was evaluated at 24, 48, and 72 h post-treatment by examining the parasites under a light microscope at 40× magnification for at least 1 min, using visible light to stimulate their movement. Based on their mobility, the parasites were classified into four categories: healthy, slightly affected (showing reduced movement), affected (exhibiting significantly reduced or abnormal movement), and dead (no observable movement) [26].

### 2.4. Scanning Electron Microscopy (SEM) on Parthenogenetic Females of Strongyloides venezuelensis

The effect of chalepensin and graveoline on adults of *S. venezuelensis* was observed by scanning electron microscopy (SEM). After 72 h of incubation, *S. venezuelensis* adults of each group of in vitro experiment were removed and fixed by immersing them immediately in 2.5% glutaraldehyde at room temperature for 3 h. The samples were then washed three times with 0.1 M phosphate buffer (pH 7.4) at room temperature for 30 min. After the final wash, they were stored at 4 °C. Subsequently, the samples were attached to Poly-L-Lysine in a humid chamber with 25% glutaraldehyde and incubated overnight. The next day, they were rinsed and fixed with 1% osmium tetroxide. After dehydration with alcohols, critical-point drying was performed, followed by metal coating. The process was conducted at the Electron Microscopy Facilities–NUCLEUS of the University of Salamanca, and visualization was carried out using a Jeol JSM-IT500 InTouchScope™ SEM (JEOL Ltd., Tokyo, Japan), SEM images were processed using the software Jeol v1250.

### 2.5. Cytotoxic Activity of Compounds in Vero Cells

African green monkey kidney epithelial cells (Vero, ATCC CCL-81) were used in this study. These cells were maintained in Dulbecco’s Modified Eagle Medium (DMEM; Gibco, Grand Island, NE, USA) supplemented with 10% FBS and 1% antibiotic and antimycotic solution (Life Technologies, Carlsbad, CA, USA). Cultures were incubated at 37 °C in a 5% CO_2_ atmosphere with 95% relative humidity.

Vero cells (1 × 10^4^ cells per well) were seeded into transparent 96-well flat-bottom polypropylene microplates (Corning Incorporated, Corning, NY, USA). After 24 h of incubation, the cells were treated with compounds at final concentrations ranging from 10 µM to 6 mM. Untreated culture medium with and with DMSO 1% was used as control. The cells were incubated for 48 h at 37 °C in a 5% CO_2_ atmosphere. Cell viability was determined using the colorimetric 3-[4,5-dimethylthiazol-2-yl]-2,5-diphenyltetrazolium bromide (MTT) assay (Affymetrix, Cleveland, OH, USA). Formazan crystals were dissolved in 100 µL of DMSO, and the optical density (OD) was measured at 570 nm using a microplate reader (MULTISKAN GO; Thermo Fisher Scientific, Waltham, MA, USA) [23]. The percentage of cell growth inhibition was calculated as follows (1):

(1) $$\%\;Cell\;growth\;inhibition=100-\left(\left(\frac{{DO}_{570}\;Treated\;cells}{{DO}_{570}\;Untreated\;cells}\right)\times 100\right)$$

In this study, Vero cells were employed as a mammalian cell model to assess the nonspecific cytotoxicity of the compounds. The selectivity indices (SIs) were calculated to know parasitic activity in relation to the toxicity in normal mammalian cells, where a high SI indicates strong parasitic activity with minimal toxicity to the cells [27]. The SI was determined by dividing the half-maximal inhibitory concentration (IC_50_) in Vero cells by the mean lethal concentration (LC_50_) for *S. venezuelensis*, using the following Formula (2):

(2) $$SI=\frac{{IC}_{50}\;Vero\;cells}{{LC}_{50}\;S.\;venezuelensis}$$

### 2.6. Statistical Analysis

All statistical analyses were performed using the GraphPad Prism 8 software (GraphPad Software Inc., San Diego, CA, USA). The data presented here represent the means ± standard deviations (SDs) of triplicates obtained from at least three independent experiments, with a 95% confidence level. A non-regression analysis was used to calculate IC_50_ and LC_50_ values. Significant differences among different conditions were studied, using a one-way analysis of variance (1 way ANOVA) or t-test after homoscedasticity and normality studies. Post hoc Dunnett’s analyses were used to determine specific differences between groups and control ivermectin. Statistical differences were considered when *p* < 0.05.

## 3. Results

### 3.1. Compounds Isolated from Ruta chalepensis

**Figure 2:**
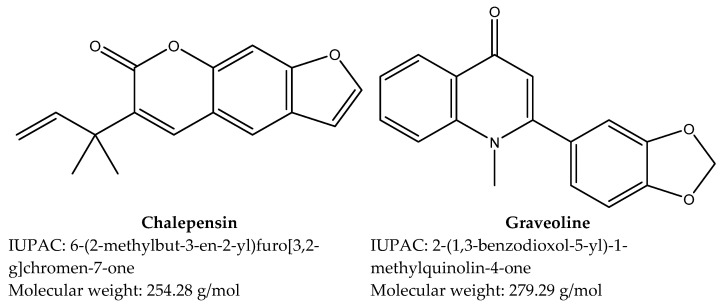
Structure of isolated compounds chalepensin and graveoline from *Ruta chalepensis* determined by spectrometric analysis.

Two compounds were successfully isolated from *R. chalepensis*, and their chemical structures were determined (Figure 2). The nematocidal activity of these compounds was evaluated against *S. venezuelensis* L3 and adults.

### 3.2. In Vitro Toxicity Against Vero Cells and Nematocidal Activity of Compounds Against Strongyloides venezuelensis L3

Nematocidal activity of the compounds against L3 of *S. venezuelensis* were tested at 24, 48, and 72 h and toxicity to Vero cells at 72 h (Table 1). The compounds were minimally toxic to Vero cells, and a slight time-dependent effect can be observed in both compounds against the infective L3. Both compounds showed good antiparasitic activity; however, the activity of chalepensin was 5 to 7 times lower than that of graveolin, depending on the incubation time, and was always similar than that of ivermectin (*p* > 0.05).

### 3.3. In Vitro Nematocidal Activity of Compounds in Strongyloides venezuelensis Adults

In Table 2, the results of the nematocidal activity of the compounds against female adults of *S. venezuelensis* at 24, 48, and 72 h are shown. Both compounds exhibited good antiparasitic activity with no time-dependent effect; at 24 h, they already show activity, which does not increase over time. In adults, as in larvae, chalepensin showed greater activity than graveoline. Furthermore, the antiparasitic effect observed against adults was lower than that obtained against larvae. In contrast, graveoline exhibited similar activity in both larvae and adults.

In Table 3, the status of the adults at different concentrations evaluated at 72 h is shown. With chalepensin, 100% mortality was observed at a dose of 30 µM, whereas with graveoline, a higher dose of 60 µM was required to achieve the same effect. In the control drug ivermectin at 10 µM, the parasites were slightly affected; however, with chalepensin at a lower dose of 3.75 µM, the same effect was observed.

**Figure 3:**
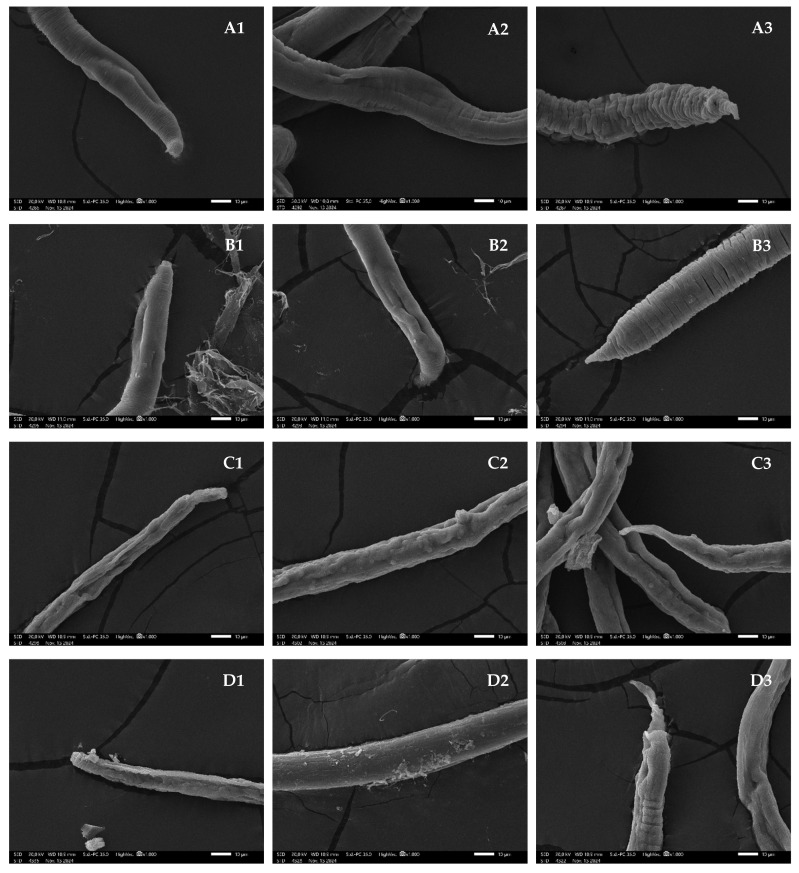
SEM images at 1000× magnification (scale = 10 µm) of *S. venezuelensis* parthenogenetic adult females with different treatments at 72 h. (**A**) Control; (**B**) ivermectin at 10 µM; (**C**) chalepensin at 30 µM; (**D**) graveoline at 60 µM. In each picture cephalic (1), body (2) and caudal (3) parts are depicted in parasites in the different treatment conditions.

The SEM images revealed distinct morphological alterations in adult parasites treated with compounds compared to the control at 72 h. In the control group, an intact cuticular structure was observed (Figure 3(A1,A2)), along with the characteristic folded morphology in the posterior region of the parasite (Figure 3(A3)). In contrast, chalepensin primarily induced the formation of numerous protuberances distributed along the entire surface of the parasite (Figure 3(C2)), accompanied by slight cuticle detachment (Figure 3(C1,C3)). Similarly, graveoline predominantly triggered cuticle detachment (Figure 3(D2)), suggesting a different mode of interaction with the parasite’s external structure. Notably, these specific alterations were absent in the ivermectin-treated group (Figure 3(B1–B3)), implying that chalepensin and graveoline may exert their antiparasitic effects through a mechanism distinct from that of ivermectin. Furthermore, despite their mechanistic differences, both compounds, along with ivermectin, induced observable morphological modifications, particularly in the posterior region of the parasite, reinforcing their potential impact on parasite viability and structural integrity.

## 4. Discussion

In this study, the antiparasitic potential of two major compounds from *Ruta chalepensis* was explored. Among the major compounds of *R. chalepensis* are chalepin, chalepensin, rutamarin [28], psoralen, bergapten [29], and graveoline [23]. In this study, the compounds evaluated were chalepensin and graveoline.

Chalepensin has been isolated from various plants, particularly those belonging to the *Rutaceae* family. It has shown in vitro activity against *E. histolytica* [16]; however, this is the first report of its antiparasitic activity in more complex organisms, such as helminths. Against *E. histolytica* trophozoites, chalepensin exhibits an LC_50_ of 180.77 µM; in comparison, our results indicate a 53-fold and 11-fold lower LC_50_ for larvae and adults of *S. venezuelensis*, respectively, highlighting its antinematicidal potential. This compound has demonstrated not only antiprotozoal activity but also antimicrobial properties, cytotoxic effects against several cancer cell lines, calcium antagonism, inhibition of platelet aggregation, and a mechanism-based inhibition of the cytochrome P450 (CYP) 2A6 enzyme [30]. Similarly, graveoline is also found in plants of the *Rutaceae* family. This compound exhibits a wide range of pharmacological effects, including antibacterial activity, spasmolytic properties, and antitumor potential [31], but no antiparasitic activity has been reported to date.

There are already some reports on the activity of plant-isolated compounds against *S. venezuelensis* larvae. For example, a study evaluated the activity of various compounds isolated from *Macleaya cordata* and *Chelidonium majus*, where the most active compound from *M. cordata* was protopine, with an LC_50_ of 33 μM, while from *C. majus* it was dehydrocorydaline, with an LC_50_ of 12 μM. However, upon determining the SIs using HL60 cells, these compounds showed very low SIs, being >0.3 and 1, respectively. Nevertheless, other compounds with higher IC_50_ values also demonstrated significantly higher SI. This was the case for oxysanguinarine and L-tetrahydrocolumbamine, both with SIs > 500 [32]. In contrast, in this study, the evaluated compounds yielded better results, with chalepensin being far superior, presenting an LC_50_ of 3.4 μM and an SI of 990 using Vero cells, and graveoline an LC_50_ of 24.4 μM and an SI of 26.2.

This study also evaluated the activity against *S. venezuelensis* adults. Few studies report activity at this stage of the parasite; most focus only on larvae. However, evaluating adults is highly relevant since they are responsible for making the parasitic infection persistent by continuously producing eggs [25]. With graveoline, similar results were obtained between larvae and adults, whereas with chalepensin, the IC_50_ increased almost fivefold in adults. It has already been reported that adults are more resistant than larvae; even ivermectin is not as effective in adults as it is in larvae [26]. In this study, chalepensin showed better results than ivermectin in adults. At 10 µM of ivermectin, the parasites were only slightly affected; however, with chalepensin at a lower dose of 3.75 µM, the same effect was observed. This highlights the antiparasitic potential of this compound against *S. venezuelensis*.

The morphology of adults exposed to lethal concentrations of the compounds was also analyzed, revealing several marked differences compared to healthy controls. In a study evaluating the effect of *Mentha × villosa* essential oil on *Schistosoma mansoni* adults, it was found to cause bubble lesions over the entire body of the worms, as well as damaged tegument and exposed musculature in some worms, along with tegument erosion [33]. Similarly, in the same parasite, it has been shown that allicin causes vesicle formation [34], while miltefosine induces tegument peeling [35]; which aligns with the observations made with graveoline, where tegument erosion was mainly noted, and chalepensin, where protuberances were observed along the body. With ivermectin, no significant morphological differences were observed compared to the control. This could be due to its mechanism of action, as ivermectin is a positive allosteric modulator that selectively opens glutamate-gated chloride channels in nematodes. This action leads to the inhibition of feeding by affecting pharyngeal muscles, paralysis by impacting motor nerves, blockage of egg release, and loss of host immunosuppression by preventing the opening of the excretory/secretory pore [36]. In contrast, the mechanism of action of chalepensin and graveoline, as they induce cuticular disruptions, may be more similar to that of praziquantel, which acts as a calcium agonist, increasing calcium concentration inside parasite cells, which leads to tegument disruption. Additionally, it exhibits synergy with the host immune system by exposing parasite antigens, allowing antibodies to recognize the parasite and activate the immune response [37].

The results of this study highlight the nematocidal activity of the compounds chalepensin and graveoline. However, it is essential to evaluate them in an in vivo murine model to confirm their antiparasitic activity and rule out any potential toxicity, as well as to conduct further assays to elucidate its mechanism of action.

## 5. Conclusions

This study demonstrated the antiparasitic effect of chalepensin and graveoline, isolated from *Ruta chalepensis*, against *Strongyloides venezuelensis* third-stage larvae and adults. Both compounds exhibited nematocidal activity and selective toxicity against parasites compared to mammalian cells, highlighting their potential as alternative treatments. Additionally, they induced morphological changes in the parasites, particularly in their cuticle, suggesting a possible mechanism of action. These findings indicate that chalepensin and graveoline possess promising antiparasitic potential, with chalepensin emerging as a particularly potent candidate for further investigation.

## Figures and Tables

**Table 1:** Mean and standard deviation of in vitro Vero toxicity at 72 h IC_50_, nematocidal activity of compounds LC_50_ in *Strongyloides venezuelensis* infective third-stage larvae (L3) at 24, 48, and 72 h, and selectivity index (SI) at 72 h.

Compound	Vero Cells IC_50_ (µM)	*S. venezuelensis* L3 LC_50_ (µM)			SI
		24 h	48 h	72 h	
Chalepensin	3365.9 ± 58.3	5.7 ± 1.2 ^a,^*	3.9 ± 1.3 ^a,^*	3.4 ± 0.9 ^a,^*	990.0
Graveoline	640.3 ± 26.4	28.3 ± 6.7 ^b^	25.9 ± 5.9 ^b^	24.4 ± 5.8 ^b^	26.2
Ivermectin	ND	2.1± 0.1	1.6 ± 0.3	1.5 ± 0.3	ND
ANOVA	-	F_(2,23)_ = 36.59 *p* = 0.0004	F_(2,23)_ = 45.46 *p* = 0.0002	F_(2,23)_ = 42.08 *p* = 0.0003	-
Dunnett’s comparison to ivermectin	-	^a^ *p* = 0.4878 ^b^ *p* = 0.0004	^a^ *p* = 0.6464 ^b^ *p* = 0.0003	^a^ *p* = 0.7294 ^b^ *p* = 0.0003	-

* No significant differences in comparison with ivermectin (*p* > 0.05). ^a^ Comparison between chalepensin and ivermectin. ^b^ Comparison between graveoline and ivermectin. ND: Not determined.

**Table 2:** Mean and standard deviations of in vitro nematocidal activity LC_50_ of chalepensin and graveoline against *S. venezuelensis* parasitic female adults and selectivity index (SI) at 72 h.

Compound	LC_50_ in µM			SI
	24 h	48 h	72 h	
Chalepensin	17.3 ± 3.1	17.1 ± 2.9	16.8 ± 2.4	200.4
Graveoline	27.8 ± 4.8	26.9 ± 5.1	26.5 ± 3.2	24.2
*t*-test	*p* = 0.0334	*p* = 0.0444	*p* = 0.0137	-

The *t*-test differences between compounds were considered when *p* < 0.05.

**Table 3:** Status categories of *S. venezuelensis* female adults at different concentrations of the compounds.

Compound	Concentration µM	Category
Chalepensin	30.0	All dead
	15.0	Most affected and some dead
	7.5	Slightly affected
	3.8	Slightly affected
	1.9	Healthy
Graveoline	60.0	All dead
	30.0	Most dead and some affected
	15.0	Slightly affected
	7.5	Healthy
	3.8	Healthy
Ivermectin	10.0	Slightly affected

## Data Availability

The datasets generated or analyzed during the present study are available from the corresponding authors.

## Supplementary Materials

The following supporting information can be downloaded at https://www.mdpi.com/article/10.3390/pathogens14050419/s1, Figures S1.1–S1.12: Spectroscopic analysis of chalepensin; Figures S2.1–S2.22: Spectroscopic analysis of graveoline.
