# Supplementary material for: Antiparasitic Activity of Chalepensin and Graveoline Isolated from Ruta chalepensis L.: In Vitro Evaluation Against Strongyloides venezuelensis
Source: Pathogens. 2025 Apr 25;14(5):419. doi: 10.3390/pathogens14050419 (PMC12114433; doi:10.3390/pathogens14050419)
Supplement: Supplementary file 1 [file pathogens-14-00419-s001.zip › pathogens-3558065-supplementary.pdf]

Supplementary Materials

Spectroscopic Data

1. *Chalepensin*

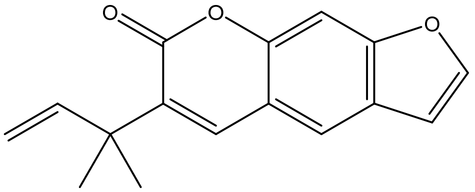

Figure S1.1. Structure of chalepensin.

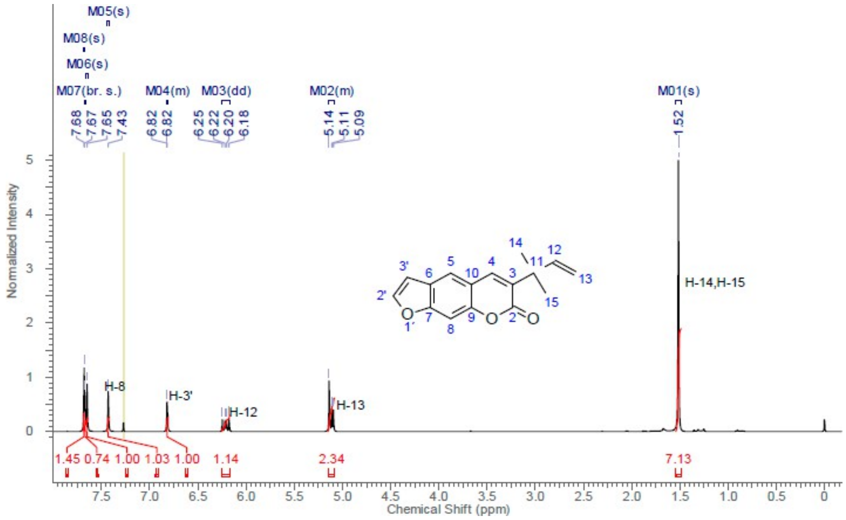

Figure S1.2. ^{1}H-NMR spectrum of chalepensin (Deuterochloroform ($CDCl_3$), 400 MHz).

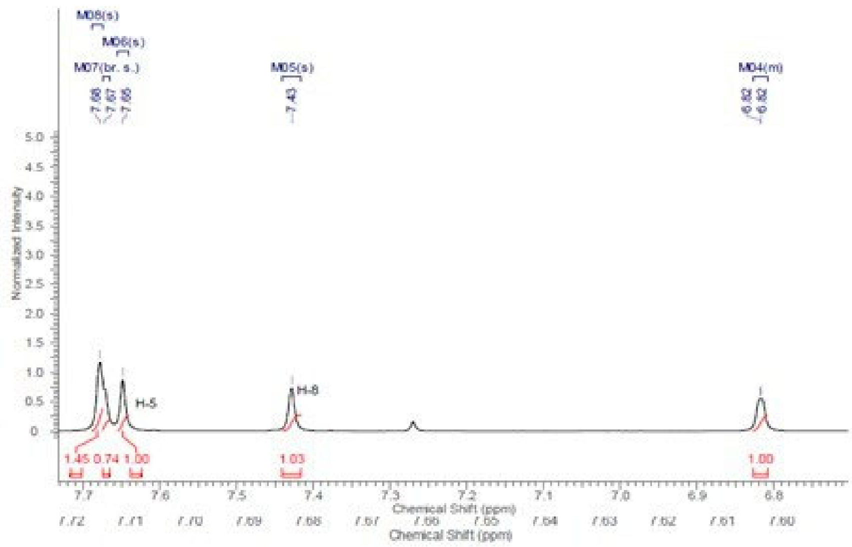

Figure S1.3. ^{1}H-NMR spectrum of chalepensin ($CDCl_3$, 400 MHz). Expansion in the area from 7.7 -6.7 ppm.

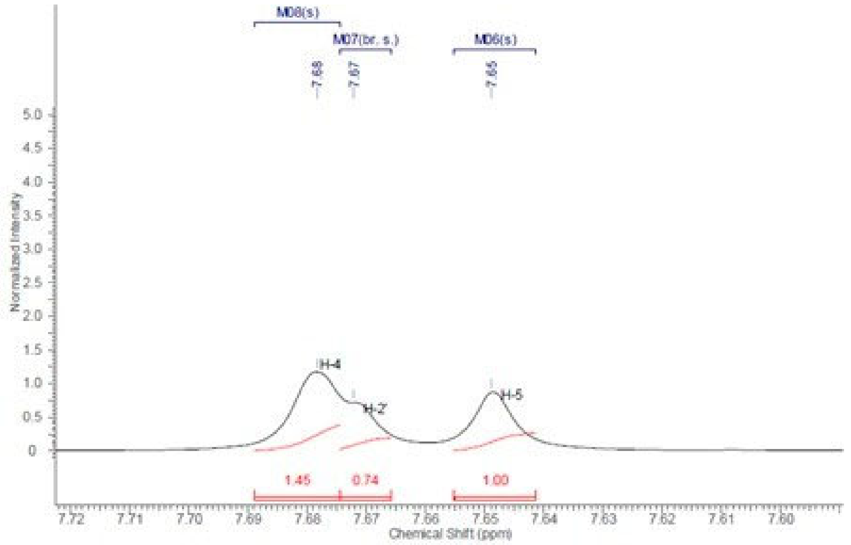

Figure S1.4. ^{1}H-NMR spectrum of chalepensin ($CDCl_3$, 400 MHz). Expansion in the area from 7.72 –7.60 ppm.

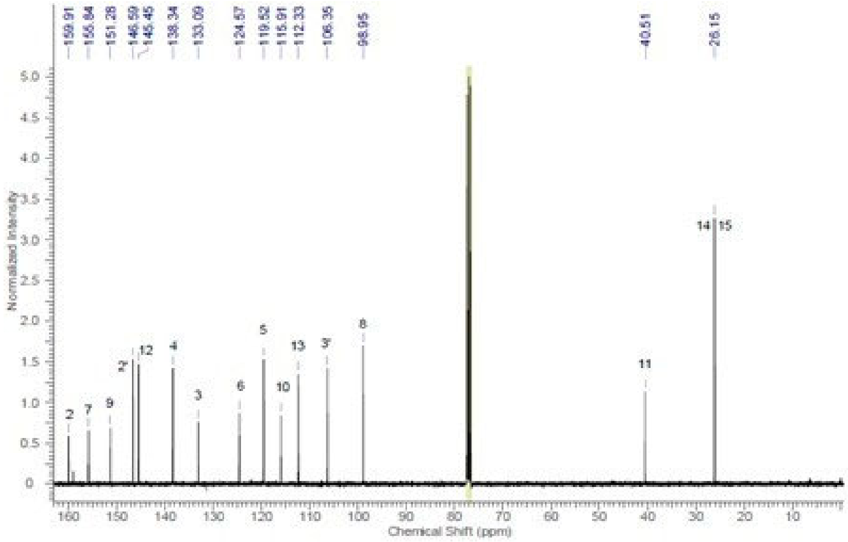

Figure S1.5. ^{13}C-NMR spectrum of chalepensin (CDCl3, 400 MHz).

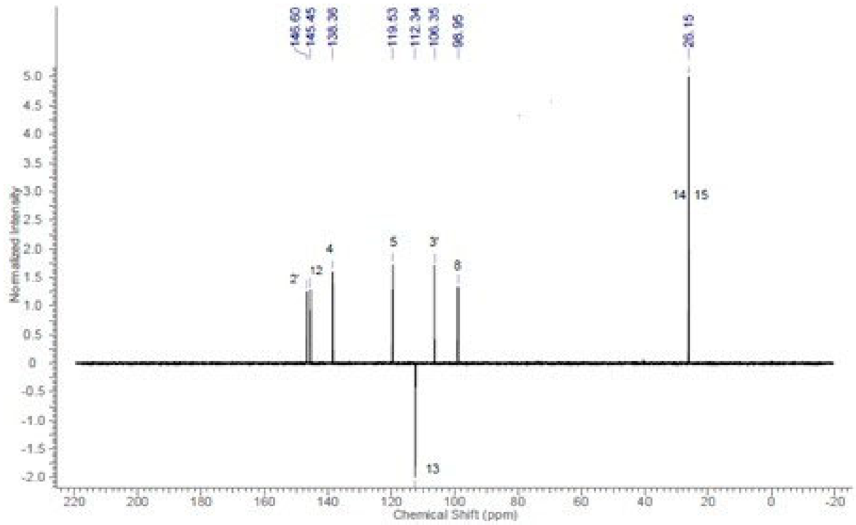

Figure S1.6. DEPT-135 spectrum of chalepensin (CDCl3, 100 MHz).

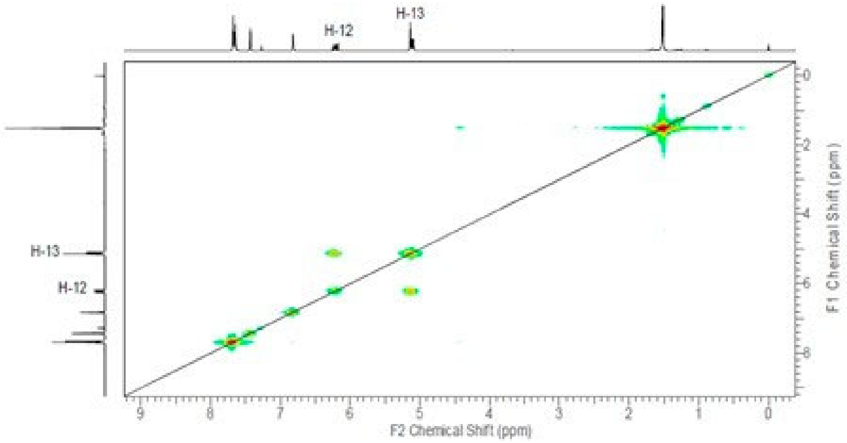

Figure S1.7. ^{1}H-^{1}H COSY spectrum of chalepensin ($CDCl_3$, 400 MHz).

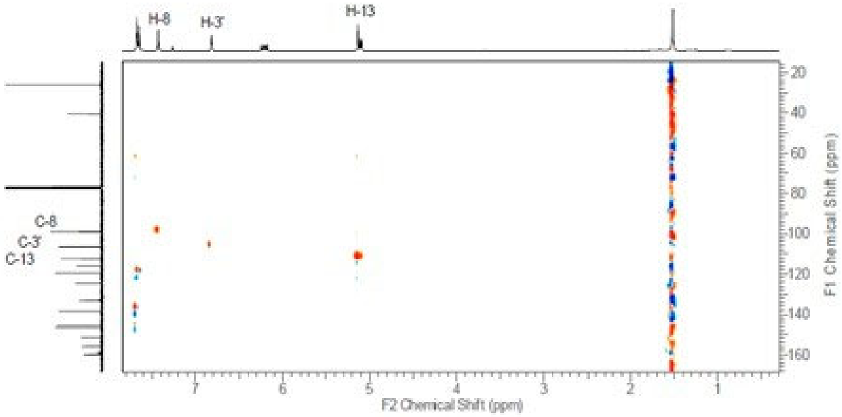

Figure S1.8. ^{1}H-^{13}C-HSQC spectrum of chalepensin ($CDCl_3$, 100 MHz).

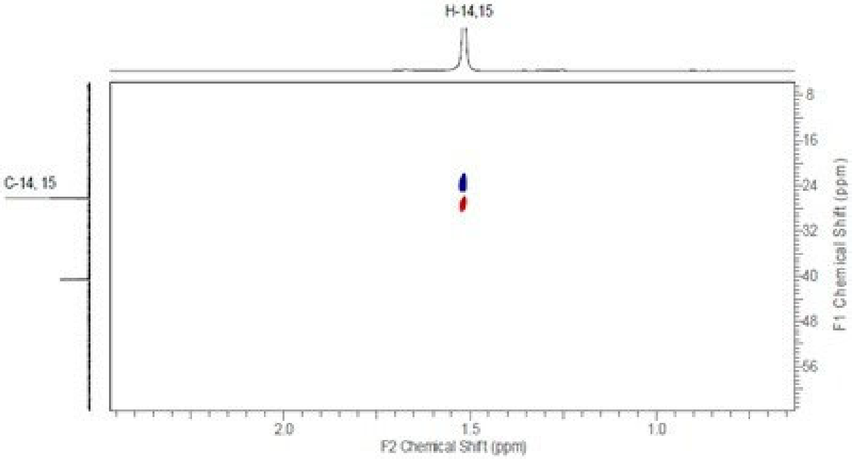

Figure S1.9. ^{1}H-^{13}C-HSQC spectrum of chalepensin ($CDCl_3$, 100 MHz). Expansion of the area ^{1}H: 0.5- 2.7 ppm, ^{13}C: 6.0 - 64 ppm.

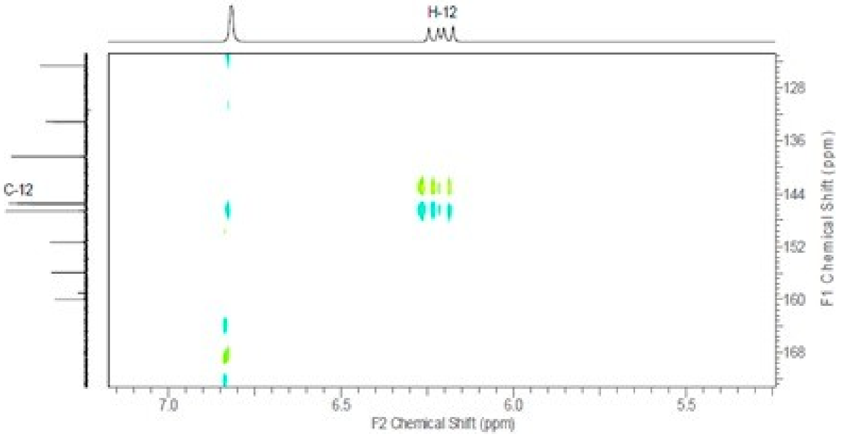

Figure S1.10. ^{1}H-^{13}C-HSQC spectrum of chalepensin (CDCl3, 100 MHz). Expansion of the area ^{1}H: 5.3- 7.1 ppm, ^{13}C: 123 - 172 ppm.

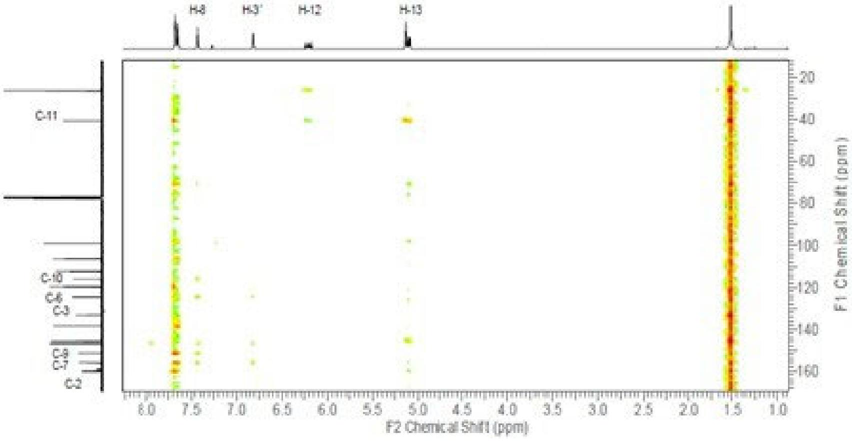

Figure S1.11. ^{1}H-^{13}C-HMBC spectrum of chalepensin (CDCl3, 100 MHz).

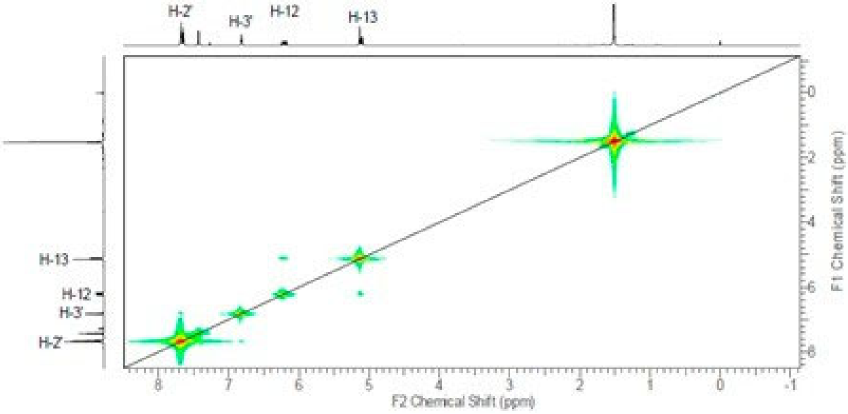

Figure S1.12. ^{1}H-^{1}H-NOESY spectrum of chalepensin (CDCl3, 400 MHz).

2. *Graveolin*

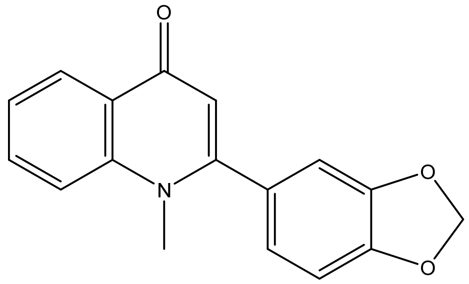

Figure S2.1. Structure of graveoline.

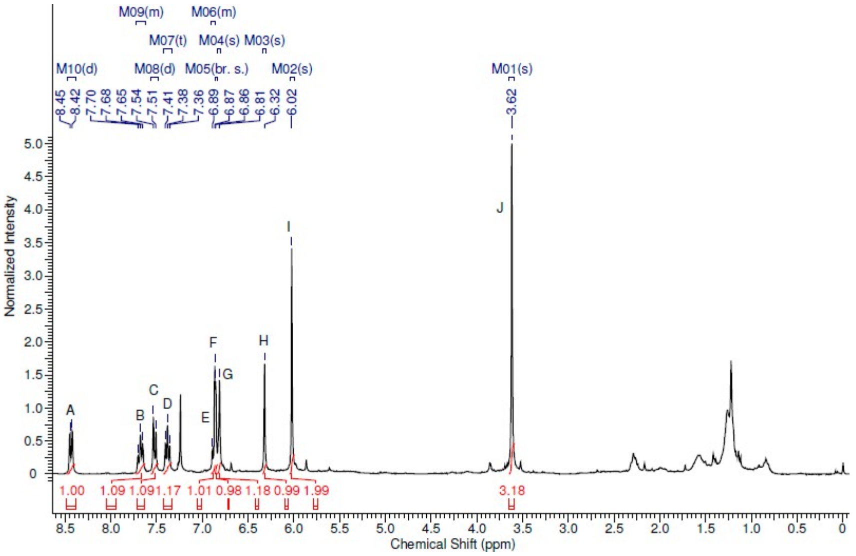

Figure S2.2. ^{1}H-NMR spectrum of graveoline ($CDCl_3$, 400 MHz).

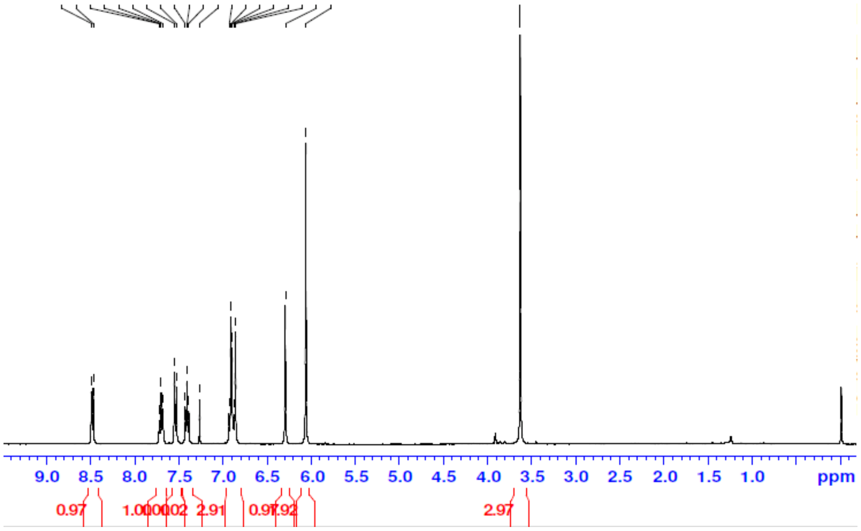

Figure S2.3. ^{1}H-NMR general spectrum of graveoline $CDCl_3$, 400 MHz).

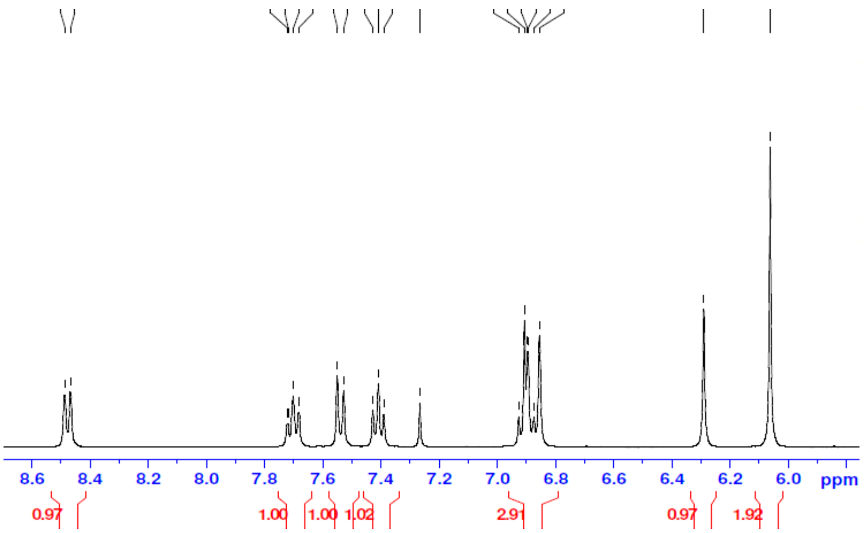

Figure S2.4. ^{1}H-NMR spectrum of graveoline ($CDCl_3$, 400 MHz). Expansion of the area ^{1}H: 5.7 – 8.7 ppm.

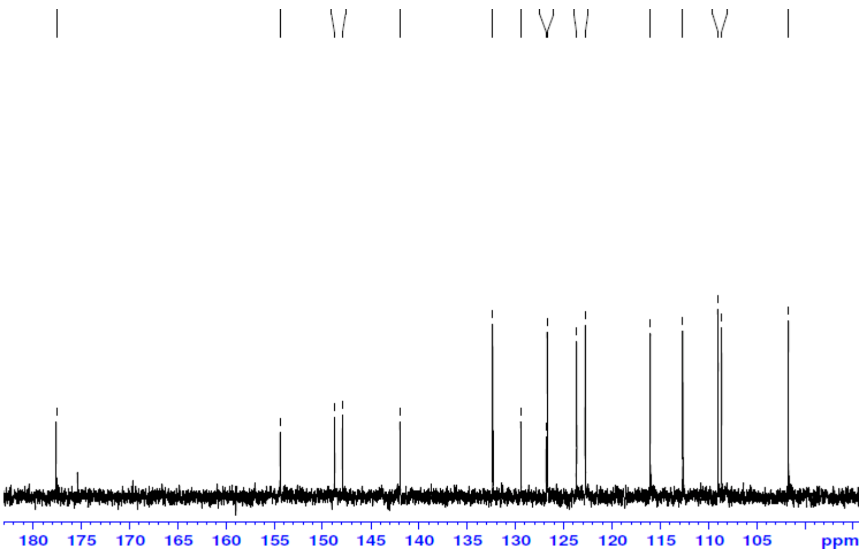

Figure S2.5. ^{1}H-NMR spectrum of graveoline ($CDCl_3$, 100 MHz). Expansion of the area ^{1}H: 94 – 183 ppm.

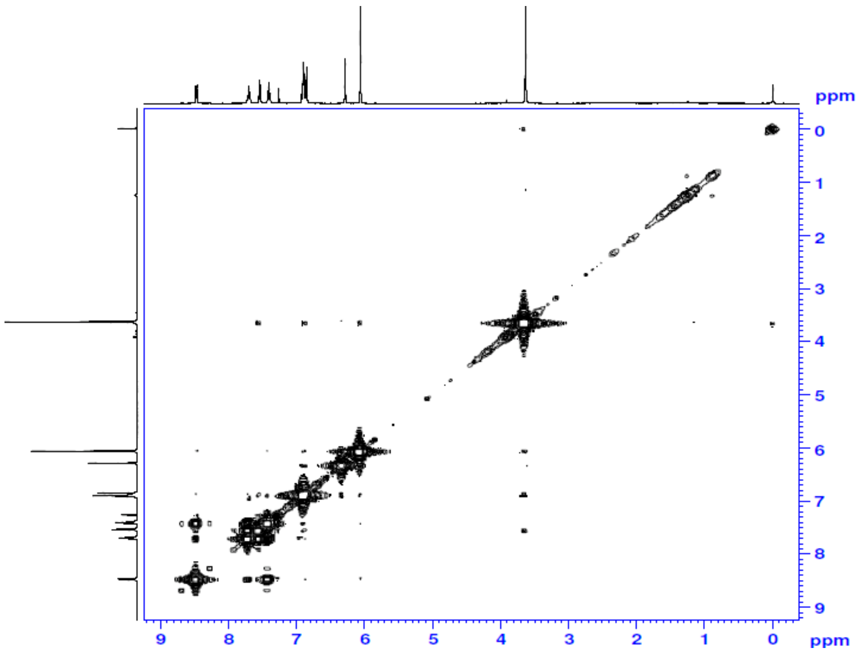

Figure S2.6 ^1H-^1H COSY general spectrum of graveoline ($CDCl_3$, 400 MHz).

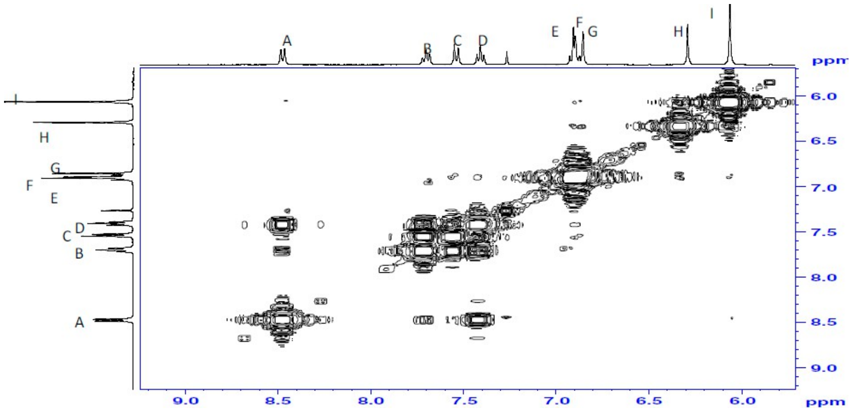

Figure S2.7. ^1H-^1H COSY spectrum of graveoline ($CDCl_3$, 400 MHz). Expansion of the area ^1H: 5.7 – 9.2 ppm, ^1H: 5.7 – 9.2.

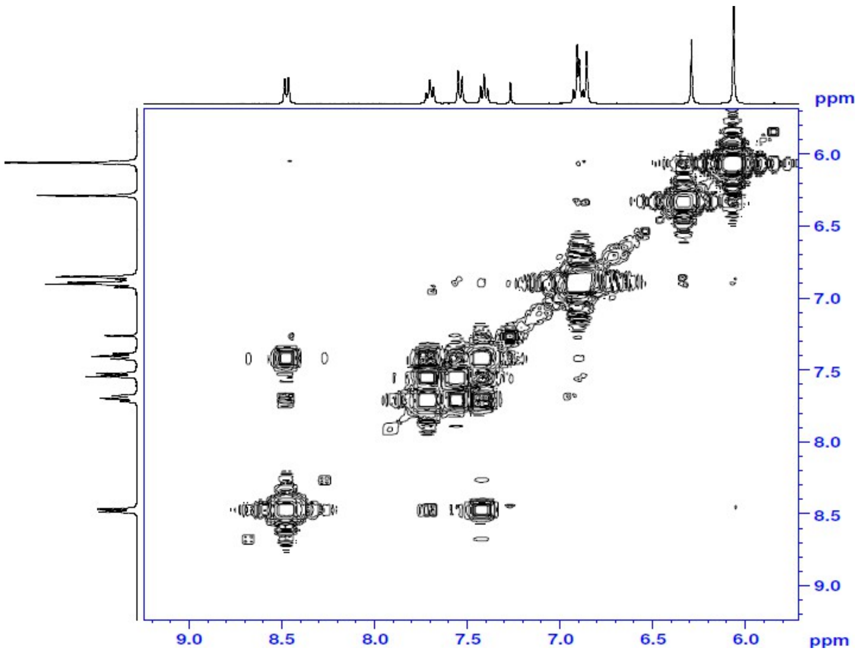

Figure S2.8. ^{1}H-^{1}H COSY spectrum of graveoline ($CDCl_3$, 400 MHz). Expansion of the area ^{1}H: 5.7 – 9.2 ppm, ^{1}H: 5.7 – 9.2.

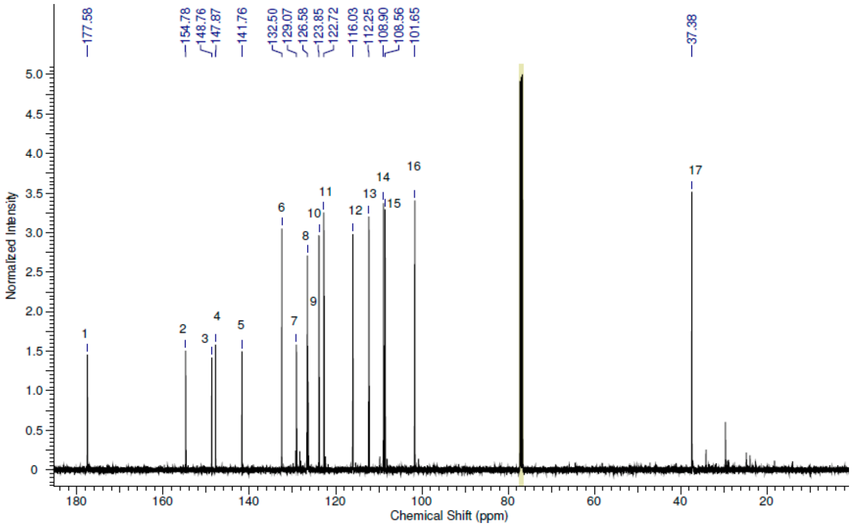

Figure S2.9. ^{13}C-NMR spectrum of graveoline ($CDCl_3$, 100 MHz).

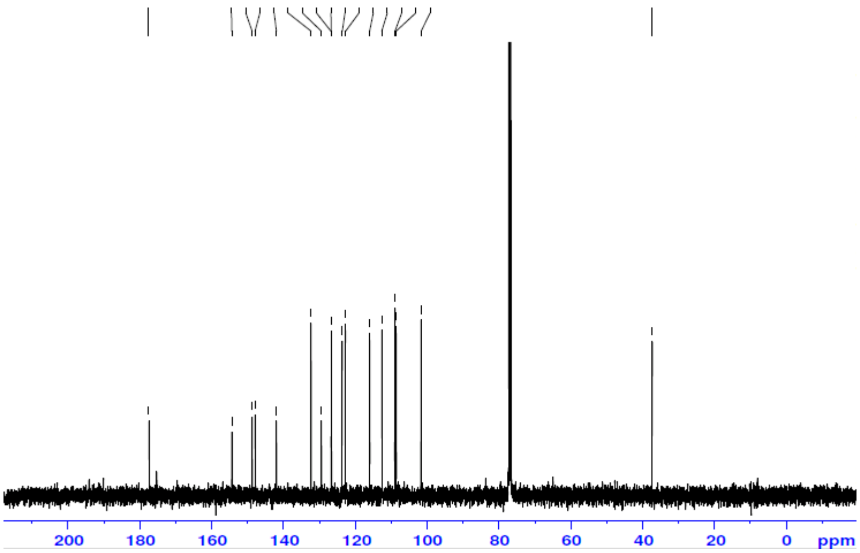

Figure S2.10. ^{13}C-NMR spectrum of graveoline ($CDCl_3$, 100 MHz).

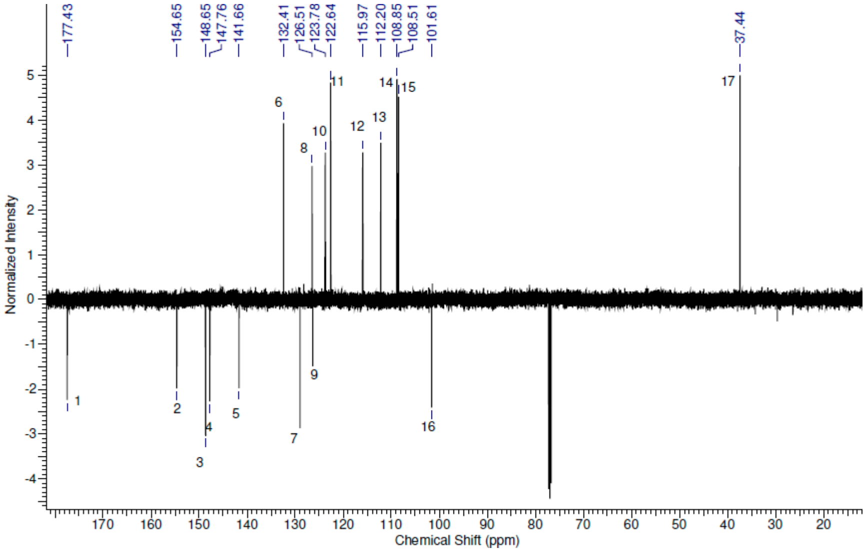

Figure S2.11. APT, ^{13}C-NMR spectrum of graveoline ($CDCl_3$, 400 MHz).

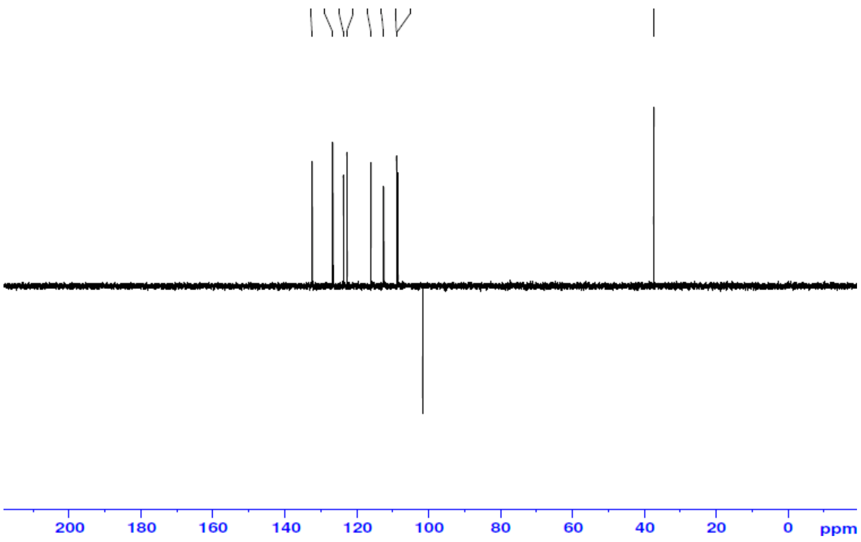

Figure S2.12. DEPT 135, ^{13}C-NMR spectrum of graveoline ($CDCl_3$, 100 MHz).

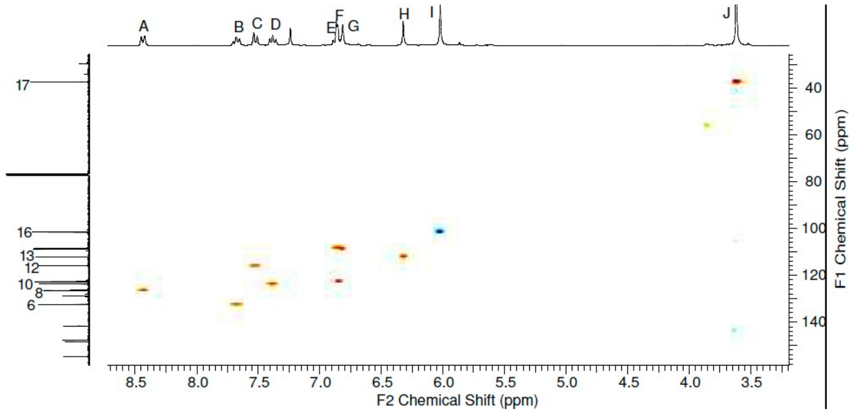

Figure S2.13. ^{1}H-^{13}C-HSQC spectrum of graveoline ($CDCl_3$, 400 MHz).

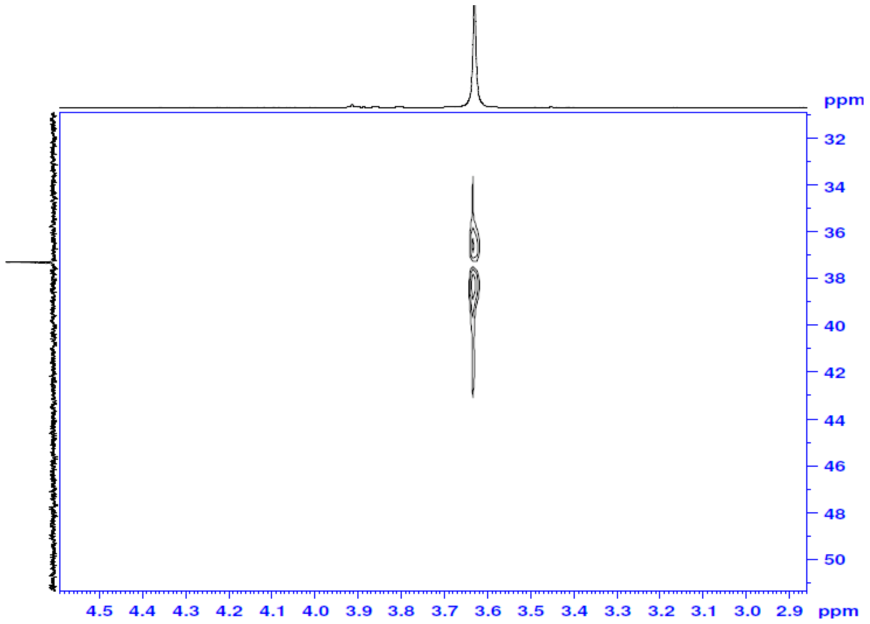

Figure S2.14. ^{1}H-^{13}C-HSQC spectrum of graveoline ($CDCl_3$, 400 MHz). Expansion of the area ^{1}H: 2.9- 4.6 ppm, ^{13}C: 31 - 51.

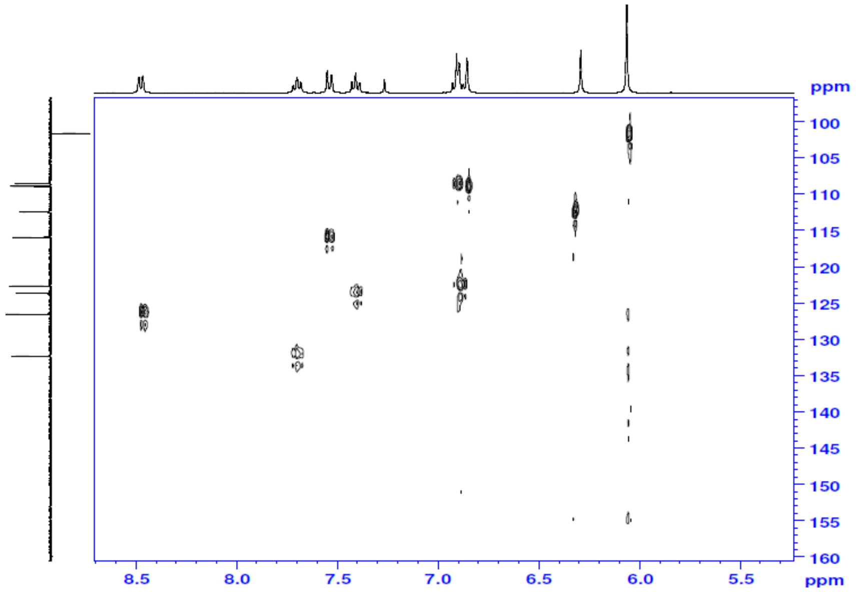

Figure S2.15. ^{1}H-^{13}C-HSQC spectrum of graveoline ($CDCl_3$, 400 MHz). Expansion of the area ^{1}H: 5.3- 8.7 ppm, ^{13}C: 95 - 160.

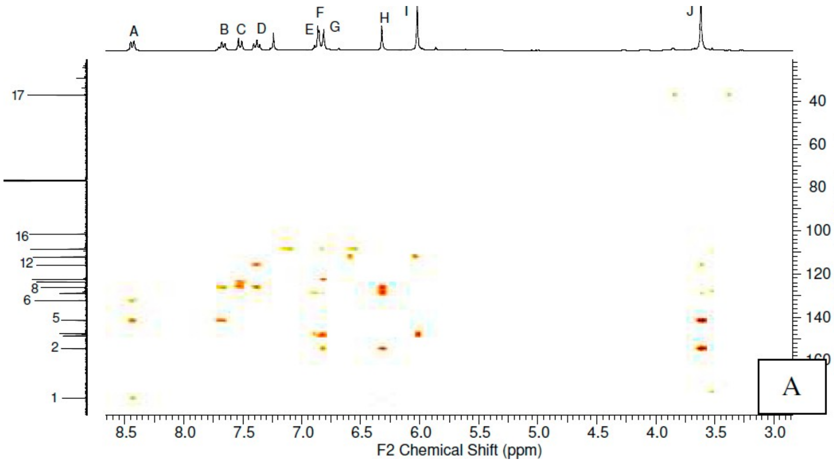

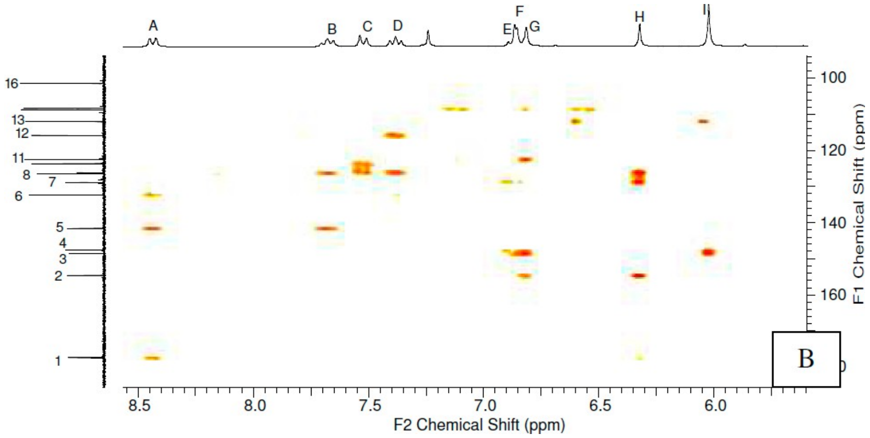

Figure S2.16. ^{1}H-^{13}C-HMBC spectrum of graveoline ($CDCl_3$, 400 MHz). A: General spectrum.; B: Expansion of the area ^{1}H: 5.0 - 8.8 ppm, ^{13}C: 90 - 185 ppm.

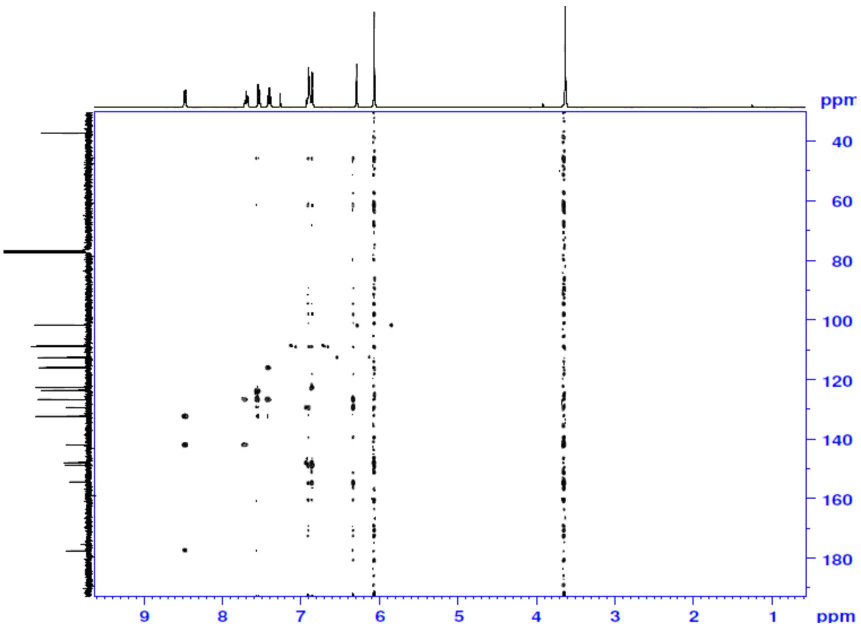

Figure S2.17. ^{1}H-^{13}C-HMBC general spectrum of graveoline ($CDCl_3$, 400 MHz).

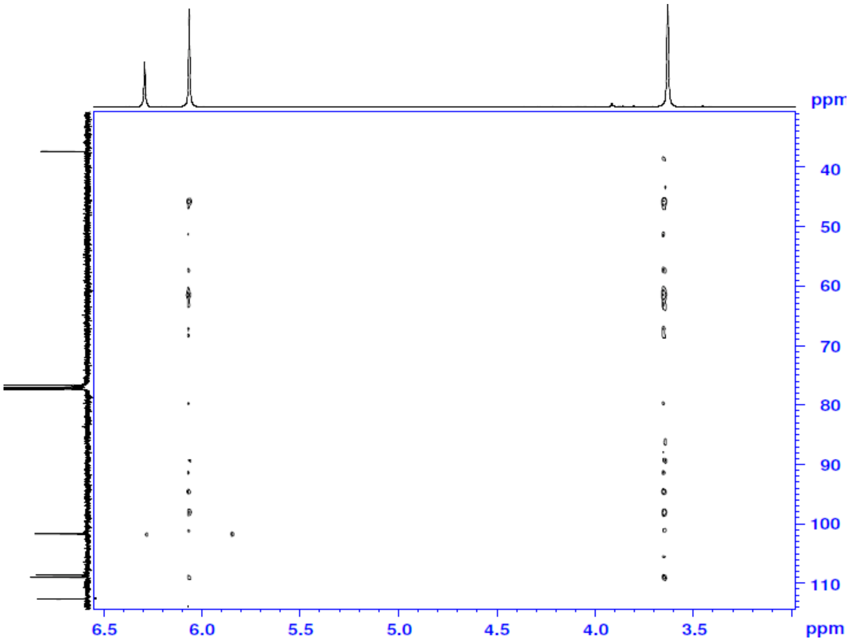

Figure S2.18. ^{1}H-^{13}C-HMBC spectrum of graveoline ($CDCl_3$, 400 MHz). Expansion of the area ^{1}H: 3.0 - 6.6 ppm, ^{13}C: 30 - 115 ppm.

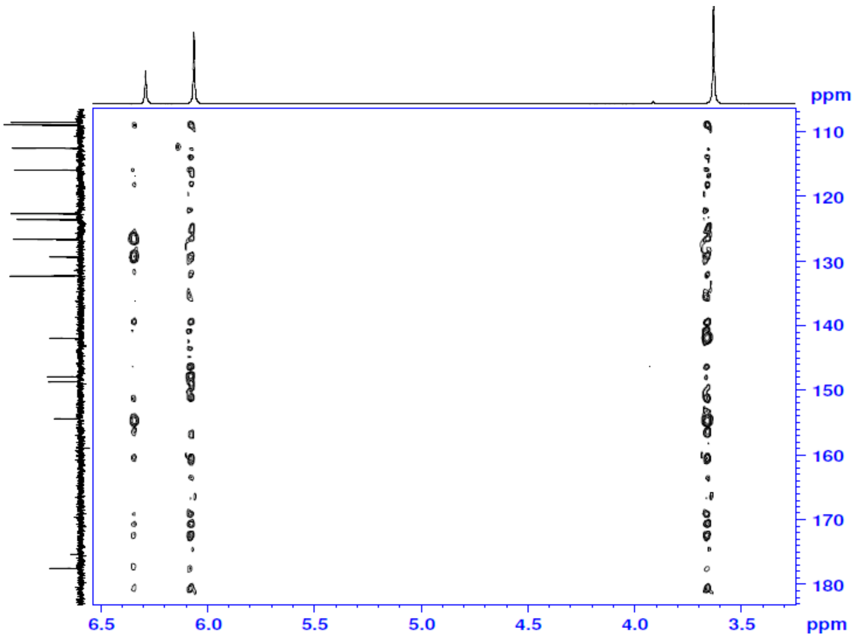

Figure S2.19. ^{1}H-^{13}C-HMBC spectrum of graveoline ($CDCl_3$, 400 MHz). Expansion of the area ^{1}H: 3.0 - 6.6 ppm, ^{13}C: 107 - 183 ppm.

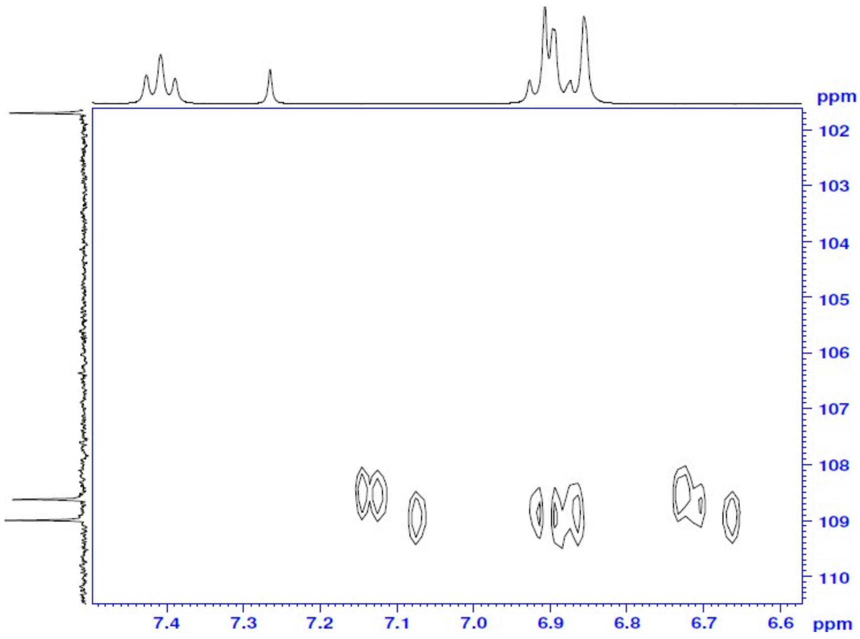

Figure S2.20 ^{1}H-^{13}C-HMBC spectrum of graveoline ($CDCl_3$, 400 MHz). Expansion of the area ^{1}H: 6.4 - 7.5 ppm, ^{13}C: 99 - 115 ppm.

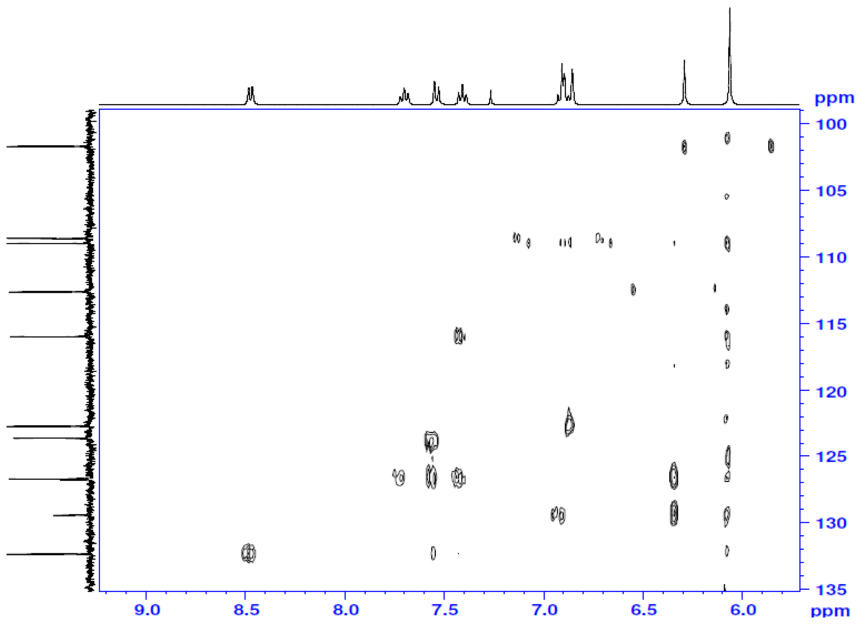

Figure S2.21. ^{1}H-^{13}C-HMBC spectrum of graveoline ($CDCl_3$, 400 MHz). Expansion of the area ^{1}H: 5.7 – 9.2 ppm, ^{13}C: 99 - 135 ppm.

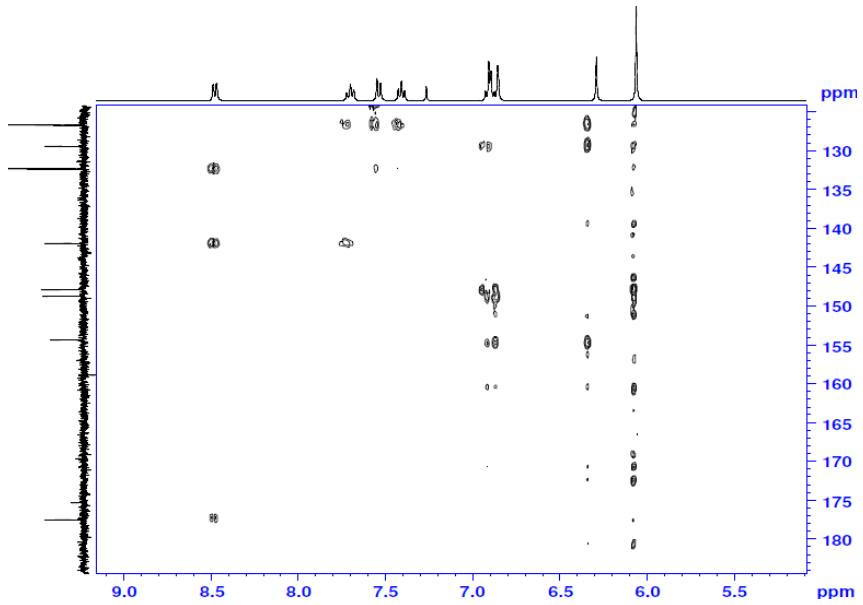

Figure S2.22. ^{1}H-^{13}C-HMBC spectrum of graveoline ($CDCl_3$, 400 MHz). Expansion of the area ^{1}H: 5.1 – 9.1 ppm, ^{13}C: 124 - 185 ppm.
